# Radiographic and echocardiographic evaluation in rescued Korean raccoon dogs (*Nyctereutes procyonoides koreensis*)

**DOI:** 10.3389/fvets.2024.1361843

**Published:** 2024-06-28

**Authors:** Junu Park, Myeongsu Kim, Jae-Ik Han, Kichang Lee, Hakyoung Yoon

**Affiliations:** ^1^Department of Veterinary Medical Imaging, College of Veterinary Medicine, Jeonbuk National University, Iksan-si, Republic of Korea; ^2^Biosafety Research Institute and College of Veterinary Medicine, Jeonbuk National University, Iksan-si, Republic of Korea; ^3^Laboratory of Wildlife Medicine, College of Veterinary Medicine, Jeonbuk National University, Iksan-si, Republic of Korea; ^4^Jeonbuk Wildlife Center, Jeonbuk National University, Iksan-si, Republic of Korea

**Keywords:** VHS, VLAS, ICS, CTR, echocardiography, reference range, Korean raccoon dog, LVIDDN

## Abstract

**Introduction:**

*Nyctereutes procyonoides koreensis* (Korean raccoon dog), a member of the Canidae family, is anatomically similar to dogs. Previous studies have used vertebral heart scale measurements to measure the cardiac size of Korean raccoon dogs on thoracic radiographs; however, the use of additional cardiac size indices, such as vertebral left arial score, intercostal space, cardiothoracic ratio, and echocardiographic indices, has not been reported. Therefore, this study aimed to establish normal reference ranges for various thoracic radiographic and echocardiographic indices in normal Korean raccoon dogs.

**Methods:**

Twenty-six Korean raccoon dogs (11 males and 15 females) were included in this study. The thoracic radiographic indices, vertebral heart scale score, and vertebral left atrial score were measured in the right lateral view. The intercostal space and cardiothoracic ratio were measured in the ventrodorsal view. The echocardiograms were evaluated in the right parasternal long and short axis view and left parasternal apical view.

**Results:**

The mean values for the thoracic radiographic and echocardiographic indices were as follows: vertebral heart scale, 9.12 ± 0.74; vertebral left atrial score, 1.5 ± 0.31; intercostal spaces, 3.17 ± 0.34; cardiothoracic ratio, 0.69 ± 0.07; left atrial to aortic root ratio, 1.22 ± 0.14; main pulmonary artery to aorta ratio, 1.22 ± 0.14; left ventricular end-diastolic internal diameter normalized for body weight, 1.36 ± 0.19; end-diastolic volume index, 51.07 ± 19.6; end-systolic volume index, 16.54 ± 7.45; the peak velocity of early diastolic transmitral flow, 73.13 ± 15.46 cm/s; and the ratio between the transmitral flow velocities and the peak early diastolic velocity, 1.77 ± 0.47. Only percent increase in the left ventricular end-systolic internal diameter was negatively correlated with body weight. The remaining indices showed no correlations with body weight.

**Conclusion:**

To the best of our knowledge, this is the first case report covering both thoracic radiographic and endocardiographic indices for Korean raccoon dogs. Thus, the thoracic radiographic and echocardiographic indices established in this study may be used to evaluate the cardiac condition of Korean raccoon dogs.

## 1 Introduction

The Korean raccoon dog (*Nyctereutes procyonoides koreensis*), a member of the Canidae family, is a common wild animal in Korea (1). Although rare, previous study has reported atrial septal defect in Korean raccoon dog (2). A previous cardiac study on Korean raccoon dogs with a cardiac focus reported an atrial septal defect, detailing only septal defect diameter, flow velocity, and pressure gradient; thoracic radiographic and echocardiographic indices were not mentioned (2). Various indices are needed to diagnose heart disease and evaluate heart function in Korean raccoon dogs.

Thoracic radiography has been commonly used to identify the changes in heart size and secondary lung or thoracic lesions caused by cardiac disease. It has proven especially useful for the evaluation of cardiac disease in dogs (3–6). Vertebral heart scale (VHS) (3–6), vertical left arial score (VLAS) (7–9), intercostal space (ICS) (3), sternal contact (10), and cardiothoracic ratio (CTR) (10, 11) are cardiac indices that have been used in dogs. Among these indices, only VHS (9.03 ± 0.52) at right lateral position, but not VLAS, ICS, sternal contact, and CTR has been used in Korean raccoon dogs (1).

Echocardiography remains the gold standard examination for the diagnosis of cardiac disease and the evaluation of cardiac function (12–18). The left atrial to aortic root ratio (LA:Ao ratio), left ventricular end-diastolic internal diameter normalized for body weight (LVIDDN), and end-diastolic volume index (EDVI) have been used to evaluate volume overload in patients with cardiac disease (19). The left atrial pressure of the heart and the diastolic function of the heart have been evaluated using the peak velocity of early diastolic transmitral flow (E wave), peak velocity of late diastolic transmitral flow (A wave), ratio between the transmitral flow velocities (E:A ratio), systolic motion (S’wave), peak early diastolic velocity (E’ wave), peak late diastolic velocity (A’ wave), and isovolumic contraction time (IVCT) is measured from the end of A’ to the beginning of S′. Isovolumic relaxation time (IVRT) is measured from the end of S′ to the beginning of E’. The time between IVCT and IVRT is defined as the ejection time (ET) and Tei index (14–16), or the ratio of E wave to IVRT (E:IVRT). The systolic function has been evaluated using the end-systolic volume index (ESVI) and fractional shortening (FS) (20, 21). Pulmonary artery deformation due to pulmonary hypertension or intrapulmonary eddy currents has also been evaluated. Measurement of the main pulmonary artery to aorta ratio (MPA:Ao ratio), right pulmonary artery distensibility index (RPAD index), tricuspid annular plane systolic excursion (TAPSE) (22, 23), pulmonic valve velocity (PV) can be helpful in evaluating pulmonary stenosis, and aortic valve velocity (AV) can be used to monitor aortic stenosis (24–26).

Normal reference ranges have not been established for the identification of echocardiographic abnormalities in Korean raccoon dogs. Therefore, this study aimed to establish normal reference ranges for the thoracic radiographic and echocardiographic indices in rescued Korean raccoon dogs without administering drugs that can affect cardiac size or function, such as sedatives or anesthetics.

## 2 Materials and methods

### 2.1 Animals

In this study, Korean raccoon dogs displaying excessive agitation or aggression were excluded from the measurements, and only cooperative Korean raccoon dogs wearing muzzles were assessed. Overall, 33 Korean raccoon dogs, comprising 16 males and 17 females weighing 2.55–5.9 kg, rescued from the Jeonbuk Wildlife Rescue and Conservation Center between August 11, 2021, and June 8, 2023, were examined. Seven Korean raccoon dogs, comprising five males and two females, with pathologic regurgitation, auscultatory heart murmurs, or anatomical heart defects were excluded from the study. As the Korean raccoon dogs of this study were wild animals that had been rescued from a conservation center, their ages were unknown. Most Korean raccoon dogs presented with ectoparasites (*Sarcoptes scabiei*), hair loss, and scabs; however, the Korean raccoon dogs recovered following treatment. Four Korean raccoon dogs had undergone limb amputation due to trauma (two left forelegs, one right hind leg, and one left hind leg). Examinations were carried out after treating the dogs and providing pain relief, avoiding drugs that could alter cardiac size or function, like sedatives or anesthetics. No spinal deformities, such as hemivertebrae, which could influence VHS measurements, were found in any of the Korean raccoon dogs (27–29). Clinical laboratory tests (complete blood count, clinical chemistry analysis, and blood gas analysis) were conducted before thoracic radiography and echocardiography analysis..

### 2.2 Thoracic radiography

**Figure 1:**
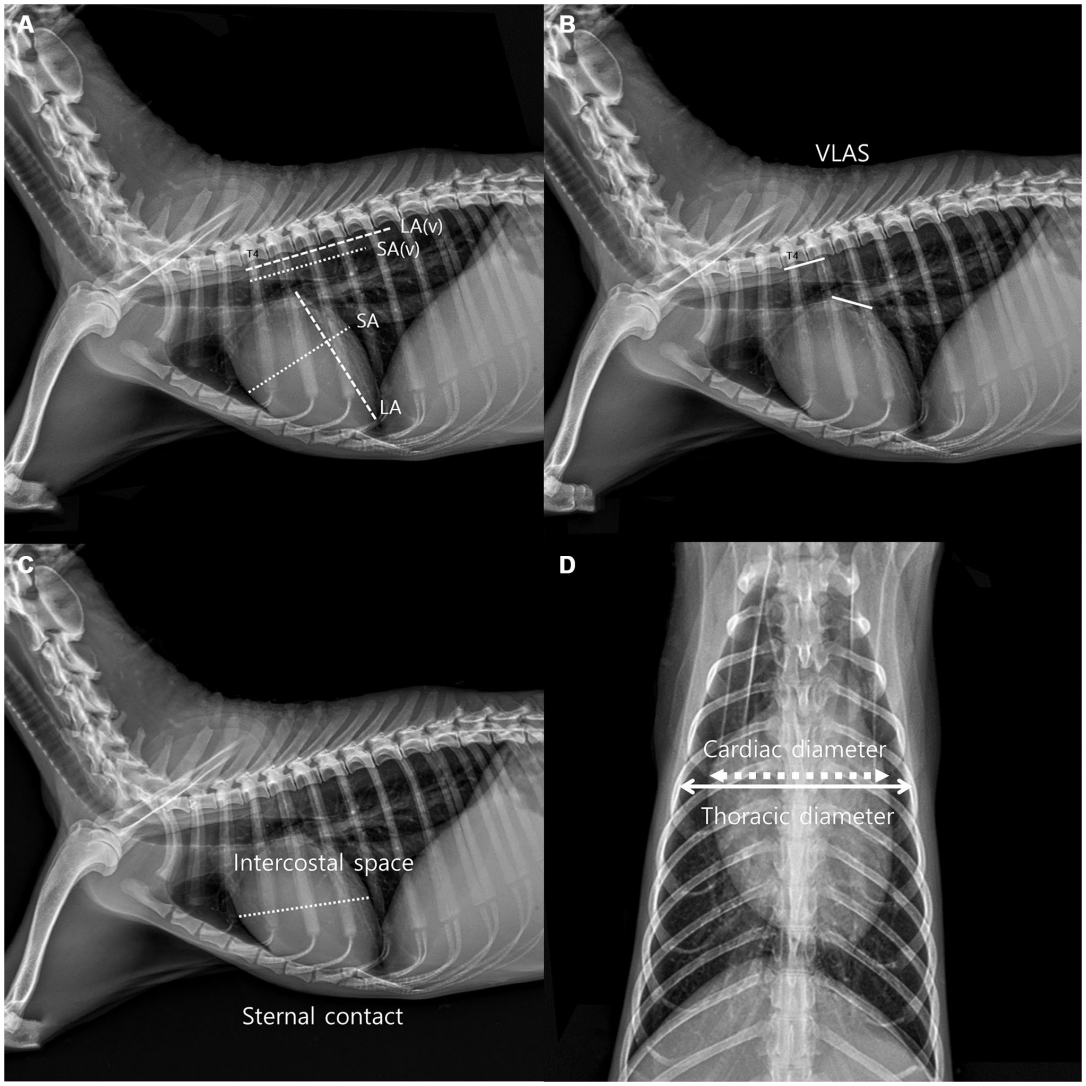
Method used to measure the vertebral heart scale (VHS). VHS was measured as per the previous measurement method used in dogs. The greatest long axis is the diameter from the center of ventral point of the carina at the trachea to the apex of the heart in the right lateral view, and the greatest short axis is the line perpendicular to the long axis. The long axis and short axis were extended toward the caudal side from the cranial edge of T4. The number of vertebrae covered in unit of 0.1 were added **(A)**. Vertebral left atrial score was also measured on the RL view from the ventral center of the carina to the most caudal side of the left atrium, where it meets the dorsal border of the caudal vena cava. The line equal to the length obtained above was extended from T4 toward the caudal side and expressed the number of vertebral body units to the nearest 0.1 vertebra **(B)**, Intercostal space (ICS), an indicator of heart size in canines and felines, was obtained by comparing the maximum diameter of the heart to the number of intercostal spaces on the lateral radiographic view. Sternal contact was measured by counting the number of intercostal spaces corresponding to the area in contact with the ventral silhouette of the heart and the sternum in the RL view **(C)**. The cardiothoracic ratio was measured on the VD view using the ratio of the thoracic width at the same level as the maximum width of the heart silhouette **(D)**.

Thoracic radiographs were acquired using a digital radiographic machine (HF-525PLUS, ECORAY, SEOUL, KOREA). VHS, VLAS, ICS, and sternal contact were measured in the right lateral view (RL view) (10), whereas CTR was measured in the ventrodorsal view (VD view) (10, 11). VHS measurements were acquired using the same technique used to acquire VHS measurements in dogs. The diameter from the center of the ventral point of the carina of the airway to the apex of the heart is defined as the greatest long axis of the heart in the RL view, whereas the line perpendicular to the long axis is defined as the greatest short axis of the heart. The long and short axes were extended caudally starting from the cranial edge of T4, and the number of vertebrae covered in 0.1 units was obtained by summing them (Figure 1A). VLAS is defined as the length from the center of the ventral point of the carina of the trachea in the RL view to the point where the caudal vena cava meets the caudal border of the left atrium. This line was extended caudally from the cranial edge of T4 and the number of vertebrae covered in 0.1 units was subsequently summed (8) (Figure 1B). ICS, which has been used as an index of cardiac size in dogs and cats, compares the maximum diameter of the heart with the number of intercostal spaces in the RL view (Figure 1C). Sternal contact is defined as the number of intercostal spaces corresponding to the area where the ventral silhouette of the heart meets the sternum in the RL view (Figure 1C) (10). CTR is defined as the ratio of the maximum width of the cardiac silhouette to the width of the thorax at the same level in the VD view (Figure 1D) (10, 11).

### 2.3 Echocardiography

**Figure 2:**
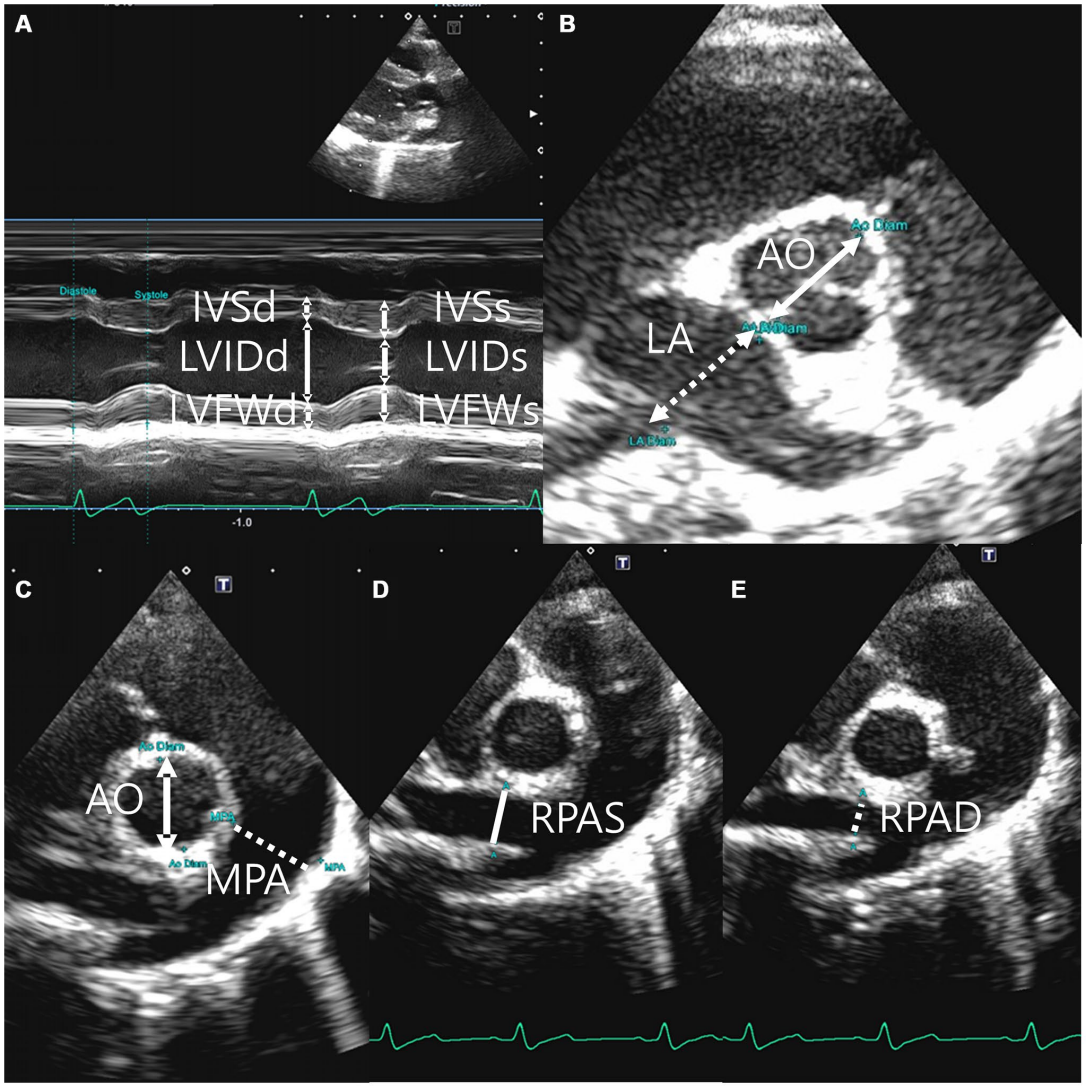
Right parasternal long axis 5 chamber view **(A)**, right parasternal short axis left atrium (La): aorta root (Ao) ratio **(B)**, right parasternal short axis main pulmonary artery (MPA): aorta root (Ao) ratio **(C)**, the minimum diastolic internal diameter of right pulmonary artery (RPAD) **(D)**, and the maximum systolic internal diameter of right pulmonary artery(RPAS) **(E)**.

Echocardiography was performed using a 5 MHz sector (Aplio 300, Canon Medical system, Europe B.V., Zoetermeer, Netherlands) (Aplio i800, Canon Medical Systems, Tokyo, Japan). The fur from the fourth to last intercostal space was shaved on both sides of the body prior to performing echocardiography. Two-dimensional, M-mode, Doppler echocardiography was performed in this study. Images were acquired in the right parasternal long axis 4 and 5 chamber view and short axis view and left parasternal apical 4 and 5 chamber view. All measurements were performed on the right and left lateral recumbency position and using the leading edge method in accordance with the American Society of Echocardiography recommendations (30, 31). A digital imaging program (Infinitt Vet PACS, Infinitt Healthcare Co., Ltd. Korea) was used to perform all thoracic radiographic and echocardiographic measurements. All reported indices were measured three or more times, and the average or largest value was generally selected with the electrocardiographic recording. The cursor was positioned at the posterior level of the cordae tendinae perpendicular to the left ventricular free wall and interventricular septum in the M-mode of the right parasternal long axis and short axis views to measure the thickness of the interventricular septum and ventricular wall and the left ventricular lumen diameter (32, 33). The left ventricular diastole and systole measurements were acquired at the origin of the QRS complex and the downward point of septal motion, respectively, on the electrocardiogram (ECG) (34). The left ventricular internal dimensions at diastole and systole (LVDd and LVDs, respectively), left ventricular free wall dimensions at diastole and systole (LVFWd and LVFWs, respectively), and interventricular septal thickness at diastole and systole (IVSd and IVSs, respectively) were measured at these time points (Figure 2A). The Teicholz formula (35) was used to calculate the left ventricular volumes, such as end-diastolic volume and end-systolic volume. The LA/Ao ratio was determined in the right parasternal short axis view at the heart’s base during early ventricular diastole, one or two frames post-aortic valve closure, which forms a symmetrical three-leaf clover shape. The left auricular appendage and atrial septum were clearly visible. The aorta’s size was measured along the border between the noncoronary and left coronary cusps, and the left atrium’s size was measured along the border between the noncoronary and left coronary cusps on the same line as the aorta (Figure 2B) (33, 36). The MPA/Ao ratio was measured in the right parasternal short axis view immediately after the closure of the pulmonary artery valve (Figure 2C) (32). The right pulmonary artery distensibility index (RPAD index) was quantified using the fractional shortening of the right pulmonary artery. The RPAD index was measured from the basilar level of the right parasternal short axis on the echocardiographic view in two-dimensional mode. The minimum internal diameter of the right pulmonary artery during diastole and systole (RPAD and RPAS, respectively) was measured subsequently. The RPAD index was calculated using the following formula:

**Figure 3:**
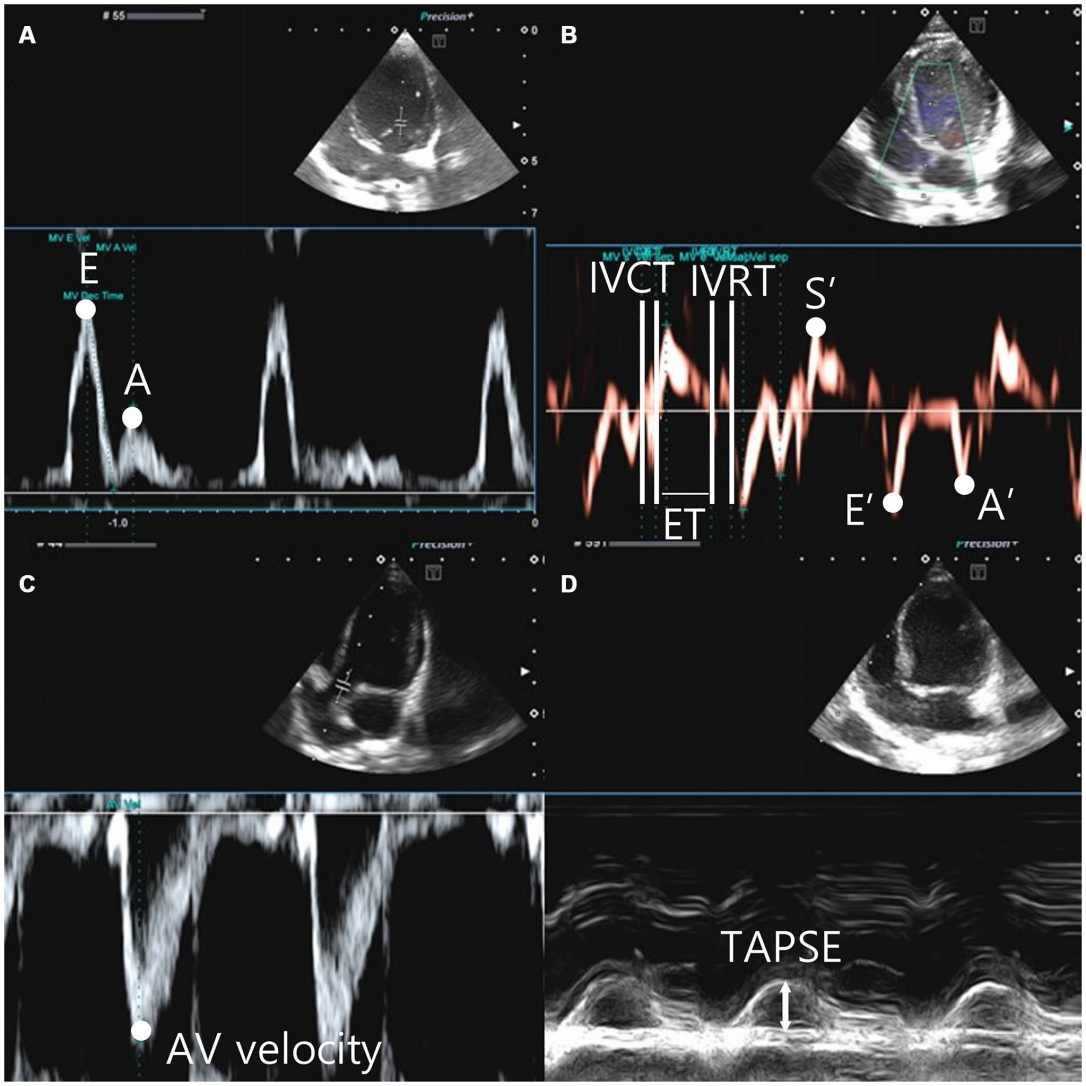
Left parasternal apical view, the peak velocity of early diastolic transmitral flow (E wave), the peak velocity of late diastolic transmitral flow (A wave) **(A)**. The tissue doppler image was gated to the annulus of the interventricular septum and the movement of the myocardium was analyzed. Systolic motion (S′), early diastolic motion (E’), late diastolic motion (A’), isovolumic contraction time (IVCT) were measured from the end of A’ to the beginning of S′. Isovolumic relaxation time (IVRT) is measured from the end of S′ to the beginning of E’. The time between IVCT and IVRT is defined as the ejection time (ET) **(B)**. Aortic velocity was measured in the left parasternal apical 5 chamber view **(C)**. Tricuspid Annular Plane Systolic Excursion (TAPSE) was measured in M-mode from left apical 4 camber view. The M-mode cursor was tuned to the tricuspid anulus site, located on the free wall of the right ventricle **(D)**.

RPAD index=([RPAS-RPAD]/RPAS)/100 (Figures 2D,E) (37). Pulse wave Doppler was used to measure the transmitral flow, early diastolic and late diastolic peak velocity (E peak and A peak, respectively), E wave deceleration time (E dec time), and E/A in the right parasternal four-chamber view (18, 38) (Figure 3A). Peak systolic velocity (S′ wave; cm/s), peak early diastolic velocity (E′ wave; cm/s), and peak late diastolic velocity (A′ wave; cm/s) from colour myocardial tissue doppler images (TDI) were acquired to determine the velocity of medial mitral annular motion at the septal portion in the left parasternal apical four-chamber view. Isovolumic contraction time (IVCT)(ms) was measured from the end of A’ to the beginning of S′. Isovolumic relaxation time (IVRT)(ms) is measured from the end of S’ to the beginning of E′. The time between IVCT and IVRT of LV is defined as the left ventricle ejection time (LVET)(ms). Tei index was calculated using the following formula: *Tei index = (IVCT + IVRT)/LVET* (14, 15, 16), ratio between E wave and IVRT (E:IVRT). The systolic function has been evaluated using the end-systolic volume index (ESVI) and fractional shortening (FS) (20, 21) ratio between the E′ waves and A′ wave, and ratio between the E wave and E′ waves were recorded (39) (Figure 3B). The formulas used in this study are as follows (28–30, 36–43):

LVIDdN = LVIDd (cm)/weight(kg)^0.294^

LVISdN = LVIDd (cm)/weight(kg)^0.294^

LVIDd inc % = [(LVIDd cm)-(1.53 × body weight (kg)^0.294^)]/(1.53 × body weight (kg)^0.294^) × 100

LVIDs inc % = [(LVIDs cm)-(1.53 × body weight (kg)^0.294^)]/(1.53 × body weight (kg)^0.294^) × 100

End-diastolic volume (EDV in ml) = 7 × LVDd^3^/(2.4 + LVDd)

End-diastolic volume index (EDVI) = EDV/ body weight (kg)^0.294^

End-systolic volume (ESV in ml) = 7 × LVDs^3^/(2.4 + LVDs)

End-systolic volume index (ESVI) = ESV/ body weight (kg)^0.294^

RWT (Relative wall thickness) = IVSd + LVFWd/LVDd

Left ventricular mass (LVM) = 0.8 × [1.04{(LVIDd cm + LVFWd cm + IVDd cm)^3^ – (LVIDd cm)^3^}] + 0.6]

Left ventricular mass index (LVMI) = LVM /body weight (kg)^0.294^

RPAD index = ([RPAS-RPAD]/RPAS)/100

Tei index = (IVCT + IVRT)/LVET

Fractional shortening (FS) = (LVDd-LVDs) × 100/LVDd

Stroke volume (SV in ml) = EDV-ESV

Ejection fraction (EF%) = (EDV-ESV) × 100/EDV

The aortic valve velocity (AV vel) was measured using pulsed wave Doppler just below the aortic valve within the left ventricular outflow tract (LVOT) in the left parasternal 5-chamber view (Figure 3C). The pulmonic valve velocity (PV vel) was measured using pulsed wave Doppler just below the pulmonic valve within the right ventricular outflow tract (RVOT) in the right parasternal short-axis view. Tricuspid annular plane systolic excursion (TAPSE) was measured in the left apical four-chamber view on M-mode. The M-mode cursor was aligned to the site of the tricuspid annulus on the free wall of the right ventricle (Figure 3D).

Mitral regurgitation (MR) and tricuspid valve regurgitation (TR) were detected at the annulus of the mitral valve (MV) and tricuspid valve (TV) using continuous waves (CW).

This study was approved by the Institutional Animal Care and Use Committee of the Jeonbuk National University, Iksan-si, Jeollabuk-do, Republic of Korea (Approval No. JBNU 2021–040).

### 2.4 Statistical analysis

All statistical analyses were performed by one of the authors (J.P.). All thoracic radiographs and echocardiographic indices were analysed using SPSS (IBM Corp., Armonk, N.Y., U.S.A.). The mean, standard deviation, and 95% confidence interval of each index were determined for the entire study population and the male and female Korean raccoon dogs separately. Pearson’s correlation test was performed as the association between body weight and each index satisfied normality. Pearson’s correlation coefficient was utilized to assess the strength and direction of the linear relationship between body weight and various indices. A positive value near 1 signifies a strong positive linear relationship, whereas a negative value near −1 indicates a strong negative linear relationship.

To determine statistically significant differences in thoracic radiographic and echocardiographic indices between males and females, a t-test was performed. Since these indices followed a normal distribution in both groups, a *p* < 0.05 was considered significant.

## 3 Results

A normal reference value was obtained for 26 Korean raccoon dogs. The average weight of the Korean raccoon dogs was 3.89 ± 0.71 kg. The average weight of the female Korean raccoon dogs was 3.79 ± 0.54 kg, whereas the average weight of the male Korean raccoon dogs was 4.01 ± 0.87 kg.

The normal references for indices obtained through thoracic radiography and echocardiography in this study were as follows:

The average values of the indices measured via thoracic radiography were as follows: VHS, 9.12 ± 0.74 (95% CI: 8.82–9.41); VLAS, 1.50 ± 0.31 (95% CI: 1.38–1.62); ICS, 3.17 ± 0.34 (95% CI: 3.03–3.31); CTR, 0.69 ± 0.07 (95% CI: 0.67–0.72); and sternal contact, 2.51 ± 0.39 (95% CI: 0.92–0.99) (Table 1).

**Table 1:** Mean, standard deviations, and 95% confidence interval of thoracic radiographic indices according to the sex and relationship between body weight.

	All		All			Sex				*p* value	Body weight	
	95% CI					Female		Male				
	Min	Max	*M*	±SD	Median	*M*	±SD	*M*	±SD		Pearson’s correlation coefficient	*p* value (*p*)
					value							
Long axis (mm)	60.54	65.36	62.95	±5.96	63.53	61.54	±6.80	64.59	±4.53	0.199	−0.081	(0.695)
Short axis (mm)	46.35	49.97	48.16	±4.47	47.86	46.99	±4.21	49.53	±4.55	0.154	−0.006	(0.977)
VHS	8.82	9.41	9.12	±0.74	9.1	8.95	±0.56	9.31	±0.89	0.225	−0.339	(0.090)
VLAS	1.38	1.62	1.5	±0.31	1.41	1.50	±0.28	1.50	±0.35	0.99	−0.226	(0.267)
ICS	3.03	3.31	3.17	±0.34	3.1	3.17	±0.23	3.17	±0.45	0.972	−0.147	(0.472)
CTR	0.67	0.72	0.69	±0.07	0.7	0.69	±0.08	0.7	±0.07	0.77	−0.094	(0.647)
Sternal contact	2.35	2.67	2.51	±0.39	2.5	2.46	±0.42	2.57	±0.37	0.52	0.235	(0.246)

^*^p < 0.05. Pearsons’s correlation coefficient: correlation between indices and body weight of the all Korean raccoon dogs. CI, confidence interval; min, minimum; max, maximum; M, mean; SD, standard deviation; VHS, vertebral heart scale; VLAS, vertebral left atrial size; ICS, intercostal space; CTR, cardiac thoracic ratio.

The mean values of indices obtained from two-dimensional and m-mode echocardiography were as follows: LA/Ao, 1.22 ± 0.14 (95% CI: 1.17–1.28); MPA/Ao, 0.96 ± 0.09 (95% CI: 0.92–0.99); LVIDd/Ao, 1.89 ± 0.32 (95% CI: 1.76–2.01); IVSd, 5.11 ± 0.85 (95% CI: 4.77–5.45); LVIDd, 20.00 ± 2.77 (95% CI: 18.88–21.12); LVFWd, 5.45 ± 0.97 (95% CI: 5.06–5.84); IVSs, 7.50 ± 0. 95 (95% CI: 7.12–7.89); LVIDs, 12.38 ± 1.35 (95% CI: 11.84–12.93); LVFWs, 8.37 ± 1.38 (95% CI: 7.81–8.92); LVIDDN, 1. 36 ± 0.19 (95% CI: 1.29–1.44); LVIDSN, 0.83 ± 0.13 (95% CI: 0.78–0.89); LVIDd inc%, −11.28 ± 12.05 (95% CI: −16.15 to 6.42); LVIDs inc%, −14.06 ± 10.34 (95% CI: −18.24 to 9.89); EDVI, 51.07 ± 19.60 (95% CI: 43.15–58.98); ESVI, 16. 54 ± 7.45 (95% CI: 13.53–19.55); RWT, 0.57 ± 0.16 (95% CI: 0.50–0.63); LVMI, 71.47 ± 15.10 (95% CI: 65.37–77.57); TAPSE (㎝), 1.61 ± 2.18 (95% CI: 0.73–2.49); and RPAD index, 0.35 ± 0.05 (95% CI: 0.33–0.37) (Table 2).

**Table 2:** Mean, standard deviations, and 95% confidence interval of two-dimensional and m-mode echocardiographic indices according to the sex and relationship between body weight.

	All		All			Sex				*p* value	Body weight		
	95% CI					F		M					
	Min	Max	*M*	±SD	Median value	*M*	±SD	*M*	±SD		Pearson’s correlation coefficient	*p* value (*p*)	
LA/Ao	1.17	1.28	1.22	±0.14	1.22	1.24	±0.14	1.20	±0.14	0.455	−0.230	(0.258)	
MPA/Ao	0.92	0.99	0.96	±0.09	0.97	0.94	±0.08	0.98	±0.10	0.235	−0.019	(0.927)	
LVIDd/Ao	1.76	2.01	1.89	±0.32	1.89	1.86	±0.25	1.92	±0.39	0.622	0.074	(0.719)	
IVSd	4.77	5.45	5.11	±0.85	5.10	5.16	±0.96	5.05	±0.73	0.739	0.040	(0.847)	
LVIDd	18.88	21.12	20.00	±2.77	20.30	19.47	±2.88	20.63	±2.63	0.300	0.236	(0.245)	
LVFWd	5.06	5.84	5.45	±0.97	5.40	5.21	±0.69	5.73	±1.19	0.186	0.186	(0.364)	
IVSs	7.12	7.89	7.50	±0.95	7.50	7.59	±0.76	7.41	±1.15	0.643	0.168	(0.411)	
LVIDs	11.84	12.93	12.38	±1.35	12.25	12.22	±1.55	12.57	±1.11	0.527	−0.065	(0.754)	
LVFWs	7.81	8.92	8.37	±1.38	8.35	8.23	±1.46	8.53	±1.33	0.596	0.195	(0.340)	
LVIDDN	1.29	1.44	1.36	±0.19	1.39	1.32	±0.18	1.42	±0.20	0.205	0.021	(0.919)	
LVIDSN	0.78	0.89	0.83	±0.13	0.83	0.81	±0.10	0.87	±0.16	0.236	−0.087	(0.674)	
LVIDd inc%	−16.15	−6.42	−11.28	±12.05	−9.37	−13.85	±11.81	−8.29	±12.12	0.249	−0.030	(0.886)	
LVIDs inc%	−18.24	−9.89	−14.06	±10.34	−15.15	−15.21	±10.55	−12.72	±10.38	0.551	−0.424	(0.031)	*
EDVI	43.15	58.98	51.07	±19.60	51.28	49.93	±17.29	52.40	±22.71	0.756	−0.218	(0.285)	
ESVI	13.53	19.55	16.54	±7.45	15.19	14.81	±4.72	18.55	±9.58	0.208	0.031	(0.882)	
RWT	0.50	0.63	0.57	±0.16	0.56	0.56	±0.11	0.58	±0.21	0.765	0.000	(0.998)	
LVMI	65.37	77.57	71.47	±15.10	70.91	67.64	±13.15	75.93	±16.55	0.168	−0.180	(0.378)	
TAPSE(cm)	0.73	2.49	1.61	±2.18	1.22	1.20	±0.36	2.09	±3.20	0.307	0.039	(0.848)	
RPAD index	0.33	0.37	0.35	±0.05	0.37	0.35	±0.04	0.36	±0.06	0.708	0.124	(0.547)	

^*^p < 0.05. Pearsons’s correlation coefficient: correlation between indices and body weight of the all Korean raccoon dogs. CI, confidence interval; min, minimum; max, maximum; M, mean; SD, standard deviation; LA/Ao, left atrium aorta ratio; MPA/Ao, main pulmonary artery aorta ratio; FS, fractional shortening; EF, ejection fraction; LVIDd/Ao, left ventricular end-diastolic internal diameter aorta ratio; IVSd, interventricular septal thickness at diastole; LVIDd, left ventricular end-diastolic internal diameter; LVFWd, left ventricular free wall dimensions at diastole; IVSs, left ventricular free wall dimensions at systole; LVFWs; LVIDDN, left ventricular end-diastolic internal diameter normalized for body weight; LVIDSN, left ventricular end-systolic internal diameter normalized for body weight; LVIDd inc%, percent increase in LVIDd; LVIDs inc%, percent increase in LVIDs; EDVI, end-diastolic volume index; ESVI, end-systolic volume index; RWT, relative wall thickness; LVMI, left ventricular mass index; TAPSE, Tricuspid Annular Plane Systolic Excursion; RPAD index, right main pulmonary artery distensibility index.

The mean values of the indices obtained from pulsed wave doppler, CW doppler, and tissue doppler echocardiography were as follows: E peak (cm/s), 73.13 ± 15.46 (95% CI: 66.89–79.38); A peak, 44.81 ± 19.29 (95% CI: 37.01–52.60); S′, 7. 86 ± 1.65 (95% CI: 7.20–8.53); E′, 7.94 ± 1.97 (95% CI: 7.14–8.73); A′, 4.99 ± 1.16 (95% CI: 4.53–5.46); IVCT, 34. 08 ± 10.90 (95% CI: 29.67–38.48); LVET, 160.08 ± 27.89 (95% CI: 148.81–171.34); IVRT, 55.85 ± 22.33 (95% CI: 46.83–64. 87); E dec time, 82.00 ± 15.85 (95% CI: 75.60–88.40); E/A, 1.77 ± 0.47 (95% CI: 1.58–1.96); E/IVRT, 1.64 ± 1. 07 (95% CI: 1.21–2.07); E/E′, 9.63 ± 2.59 (95% CI: 8.58–10.68); E′/A′, 1.68 ± 0.52 (95% CI: 1.47–1.89); Tei index, 0. 58 ± 0.34 (95% CI: 0.44–0.72); FS, 37.18 ± 8.12 (95% CI: 33.90–40.45); EF, 69.39 ± 11.08 (95% CI: 64.91–73.86); AV vel profile (cm/s), 112.72 ± 19.46 (95% CI: 104.86–120.58); and PV vel profile (cm/s), 98.29 ± 30.67 (95% CI: 85.90–110.68) (Table 3).

There were no statistically significant differences observed between male and female in any of the thoracic radiographic and echocardiographic indices.

Pearson’s correlation test revealed a negative correlation between the LVIDs inc% index and body weight, with a *p*-value was <0.05, indicating significance. The Pearson’s correlation coefficient was between −1 and 0. The *p*-value was >0.05 for the other thoracic radiographic and echocardiographic indices; thus, it was determined that no correlation was present with body weight and other thoracic radiographic and echocardiographic indices except for the LVIDs inc% index.

**Table 3:** Mean, standard deviations, and 95% confidence interval of pulsed wave doppler, continuous wave doppler, tissue doppler echocardiographic indices according to the sex and relationship between body weight.

	All		All			Sex				*p* value	Body weight	
	95% CI					F		M				
	Min	Max	*M*	±SD	Median value	*M*	±SD	*M*	±SD		Pearson’s correlation coefficient	*p* value (*p*)
E peak (cm/s)	66.89	79.38	73.13	±15.46	72.0	70.86	±17.33	75.78	±13.19	0.429	0.048	(0.817)
A peak (cm/s)	37.01	52.60	44.81	±19.29	42.10	45.89	±25.39	43.54	±9.01	0.764	0.190	(0.352)
S′ (cm/s)	7.20	8.53	7.86	±1.65	7.80	7.58	±1.42	8.19	±1.91	0.360	0.324	(0.107)
E’ (cm/s)	7.14	8.73	7.94	±1.97	7.50	7.79	±2.20	8.11	±1.73	0.687	0.086	(0.676)
A’ (cm/s)	4.53	5.46	4.99	±1.16	5.0	5.16	±1.30	4.80	±0.98	0.438	0.109	(0.598)
IVCT (ms)	29.67	38.48	34.08	±10.90	33.0	33.36	±13.32	34.92	±7.69	0.724	−0.199	(0.330)
LVET (ms)	148.81	171.34	160.08	±27.89	165.0	162.71	±20.90	157.00	±35.10	0.613	−0.162	(0.430)
IVRT (ms)	46.83	64.87	55.85	±22.33	56.0	48.07	±21.76	64.92	±20.18	0.053	−0.116	(0.573)
E dec time (ms)	75.60	88.40	82.00	±15.85	82.0	81.14	±18.41	83.00	±12.99	0.773	0.004	(0.986)
E/A	1.58	1.96	1.77	±0.47	1.70	1.76	±0.56	1.78	±0.35	0.909	−0.106	(0.607)
E/IVRT	1.21	2.07	1.64	±1.07	1.30	1.95	±1.35	1.28	±0.47	0.116	0.044	(0.832)
E/E’	8.58	10.68	9.63	±2.59	10.08	9.63	±3.05	9.63	±2.07	0.999	−0.058	(0.780)
E’/A’	1.47	1.89	1.68	±0.52	1.56	1.61	±0.65	1.76	±0.31	0.489	−0.066	(0.748)
Tei index	0.44	0.72	0.58	±0.34	0.52	0.51	±0.22	0.67	±0.44	0.263	−0.147	(0.472)
FS	33.90	40.45	37.18	±8.12	39.66	36.81	±5.91	37.60	±10.40	0.812	0.262	(0.196)
EF	64.91	73.86	69.39	±11.08	73.05	69.32	±7.17	69.47	±14.77	0.973	0.274	(0.176)
AV vel (cm/s)	104.86	120.58	112.72	±19.46	109.30	106.47	±15.29	120.03	±21.83	0.076	0.243	(0.233)
PV vel (cm/s)	85.90	110.68	98.29	±30.67	94.95	103.67	±40.10	92.02	±12.59	0.345	0.115	(0.576)

^*^p < 0.05. Pearsons’s correlation coefficient: correlation between indices and body weight of the all Korean raccoon dogs. CI, confidence interval; min, minimum; max, maximum; M, mean; SD, standard deviation; E peak, the peak velocity of early diastolic transmitral flow; A peak, the peak velocity of late diastolic transmitral flow; S′, the peak systolic velocity; E’, the peak early diastolic velocity; A’, the peak late diastolic velocity; IVCT, isovolumic contraction time; LVET, left ventricle ejection time; IVRT, isovolumic relaxation time; E dec time, E peak deceleration time; AV vel, aortic valve velocity; PV vel, pulmonary valve velocity.

## 4 Discussion

The Korean raccoon dog is a member of the Canidae family whose cardiac structure is similar to that of dogs. Cardiac disease similar to that observed in dogs has been reported in Korean raccoon dogs. A previous cardiac study involving Korean raccoon dogs reported one case of atrial septal defect (2), indicating the requirement for various indices to diagnose cardiac disease and evaluate cardiac function in Korean raccoon dogs. Thoracic radiography enables the visualization of the heart as well as lesions of the lungs, thoracic cavity, and other adjacent skeletal structures that may be affected by cardiac disease. Thoracic radiography provides indices of the overall size of the heart (4). However, evaluating the size of each chamber of the heart is difficult. VHS, ICS, and CTR have been used effectively for determining the overall heart size in dogs (3, 10, 15). In addition, VLAS has been used effectively for determining the left atrium heart size (7, 8). VHS of Korean raccoon dogs was determined to be 9.12 ± 0.74 (95% CI: 8.82–9.41) in the present study, which was similar to that of dogs, such as Maltese (9.07–9.99) (9), Shih Tzus (8.3–10.7) (44), Poodles (9.1–11.1) (45), and Yorkshire Terriers (8.7–10.7) (4). The VHS measured in this study aligns with the previous value (9.03 ± 0.52) of Korean raccoon dogs (1). However, significant differences were observed compared with those of boxers (10–13.2) (46), Boston Terriers (8.9–14.5) (44), and English bulldogs (9.3–16.1) (44). The normal range of VHS varies depending on various body sizes and body types in dogs; however, it is believed that a similar reference range was obtained for Korean raccoon dogs owing to their body type being similar to that of small dogs. The ICS of the Korean raccoon dog was determined to be 3.17 ± 0.34 (95% CI: 3.03–3.31), which is similar to the ICS of dogs (2.5–3.5) (10). The normal range of CTR for dogs is <67%. The normal range for raccoons (67–72%) is slightly greater than that for dogs (10). Sternal contact was slightly smaller in Korean raccoon dogs (2.51 ± 0.39) than that in dogs (2.5–3) (47). The present study revealed that the Korean raccoon dog has an anatomical structure similar to that of dogs. Consequently, similar reference indices can be used for Korean raccoon dogs (1). The differences in some thoracic radiography heart size indices, such as CTR and sternal contact, may be attributed to the anatomical differences in the structure of the thoracic cavity between species.

Case reports of cardiac disease in the Korean raccoon dog are rare. Moreover, echocardiographic indices have not been established for non-sedated Korean raccoon dogs. This study evaluated rescued Korean raccoon dogs using echocardiographic indices frequently used in dogs and established a reference range for the echocardiographic indices. EDVI, LVIDd inc%, LVIDd/Ao, LVIDDN, and LA:Ao are indicators used to evaluate volume overload, and the average values of these indices were similar (28). E peak velocity, E:IVRT, and E:E’ are indices related to left atrial pressure (48), and the average values of these were similar to those of dogs. ESVI, LVIDs inc%, and FS may be related to contractile force (49, 50), and the average values were similar to those of dogs. MPA:Ao, RPAD index, and TAPSE are indices related to pulmonary hypertension (51), and the average values of these were similar to those of dogs. Other echocardiographic indices were also evaluated in the same manner as in dogs.

The structure of the left ventricular chamber changes from elliptical to spherical shape due to the volumeoverload of the left ventricle. Further, it is known that the Teicholz formula does not consider this structural change, resulting in an inaccurate calculation of left ventricular volume when using M-mode. The systolic volume of the chamber calculated using the Teicholz formula may be overestimated compared with that calculated from the method using method of disks (12, 35). The Modified Simpson’s rule is a method of disks and is recommended for volume determination, as it is relatively unaffected by the structure of the ventricular chamber. While the modified Simpson’s rule may be more accurate in measuring the volume of the cardiac chambers compared to the Teicholz method, clear views of both diastole and systole are required in the sharp left parasternal 2-chamber and 4-chamber views for an accurate assessment (20, 21).

All rescued Korean raccoon dogs have a wild nature and may remain tense. Excessively agitated or aggressive Korean raccoon dogs were excluded from the measurements, but it is possible that the evaluations were not performed in a complete resting state. No drugs were administered during the examination in this study to exclude the effects of drugs. However, oral drugs, such as trazodone and gabapentin, and injectable drugs, such as butorphanol, are commonly used in excited dogs and cats (52). Oral administration of drugs would be difficult in Korean raccoon dogs due to their aggressive nature. Therefore, the use of butorphanol injection may be preferred (53), and related echocardiographic evaluation after administration of butorphanol is necessary for future study.

Pearson’s correlation test revealed a negative correlation between body weight and LVIDs inc% index, with a Pearson’s correlation coefficient of −1 < r < 0 and *p*-value of <0.05 for LVIDs inc%. LVIDs inc% is an index associated with chronic heart failure in dogs and is corrected for body weight (49). Thus, this may have been an error that occurred owing to the small sample size. Indices related to heart chamber size, such as LVIDD and LVIDS, are positively correlated with body weight in dogs (54, 55); however, no such correlation with body weight was observed in this study. This finding may be attributed to the narrow body weight range of the Korean raccoon dogs examined in this study. Therefore, further studies must be conducted in the future using various body weights.

This study revealed no statistically significant sex differences in terms of heart function-related indices, such as cardiac size, ESVI, and Tei index, in Korean raccoon dogs. Sex differences were not observed in some breeds of dogs. These differences are most noticeable when differences in individual size are observed based on sex (56–59).

Body size, body shape, thoracic radiography indices, and echocardiography indices differ depending on the species of dogs (56–59). There are six known subspecies of raccoon dogs (1), and echocardiograms may differ between subspecies. Therefore, further study of echocardiographic indices in other subspecies is necessary. Estimating the age of Korean raccoon dogs was difficult; however, it is expected that the relationship among the cardiac indices according to age will be comparable.

This study had a small sample size, so we need additional large-scale research with a large sample size is needed in the future. Although all rescued Korean raccoon dogs were disease-free and had recovered from trauma, it could not be confirmed that the Korean raccoon dogs had no history of disease. Therefore, additional studies on completely normal Korean raccoon dogs must be conducted in the future. In addition, the Korean raccoon dogs may have been in a nervous state, which may have affected their cardiac indices. Moreover, they did not cooperate during the measurement of blood pressure. Therefore, information regarding blood pressure remains lacking. Blood pressure evaluation may also be necessary at a later date. In this study, Korean raccoon dogs with cardiac disease such as myxomatous mitral valve disease(MMVD) with severe mitral regurgitation or any congenital cardiac disease causing cardiomegaly were excluded in this study. It is helpful to use the normal range of cardiac indices in normal Korean raccoon dogs to distinguish between a normal heart and an enlarged heart. However, determining the degree of cardiomegaly is difficult (60). Therefore, the further study is required to establish the cut-off value by investigating the relationship between the indices of Korean raccoon dogs with cardiomegaly and normal Korean raccoon dogs in a large sample.

To the best of our knowledge, this is the first case report covering both thoracic radiographic and endocardiographic indices for Korean raccoon dogs. This study provides thoracic radiological and echocardiographic indices for Korean raccoon dogs in the absence of any sedative medication that may affect cardiac indices. This study can be useful in future cardiac evaluation on Korean raccoon dogs.

## Data availability statement

The raw data supporting the conclusions of this article will be made available by the authors, without undue reservation.

## Ethics statement

The animal study was approved by the Institutional Animal Care and Use Committee of the Jeonbuk National University. The study was conducted in accordance with the local legislation and institutional requirements.

## Author contributions

JP: Writing – original draft, Writing – review & editing. MK: Writing – original draft, Writing – review & editing. J-IH: Writing – original draft, Writing – review & editing. KL: Writing – original draft, Writing – review & editing. HY: Writing – original draft, Writing – review & editing.

## Glossary

VHS: Vertebral heart scale
VLAS: Vertical left arial score
ICS: Intercostal space
CTR: Cardiothoracic ratio
LA:Ao ratio: Left artrial to aortic root ratio
LVIDDN: Left ventricular end-diastolic internal diameter normalized for body weight
EDVI: End-diastolic volume index
E wave: The peak velocity of early diastolic transmitral flow
A wave: The peak velocity of late diastolic transmitral flow
E:A ratio: The ratio between both transmitral flow velocities
E’ wave: The peak early diastolic velocity the peak late diastolic velocity (A’ wave)
ESVI: End-systolic volume index
FS: Fractional shortening
MPA:Ao ratio: Main pulmonary artery to aorta ratio
RPAD index: Right pulmonary artery distensibility index
TAPSE: Tricuspid annular plane systolic excursion
PV: Pulmonic valve velocity
AV: Aortic valve velocity
RL view: Right lateral view
VD view: Ventrodorsal view
ECG: Electrocardiogram
LVDd: Left ventricular internal dimensions at diastole
LVDs: Left ventricular internal dimensions at systole
LVFWd: Left ventricular free wall dimensions at diastole
LVFWs: Left ventricular free wall dimensions systole
IVSd: Interventricular septal thickness at diastole
IVSs: Interventricular septal thickness at systole
TAPSE: Tricuspid annular plane systolic excursion
RPAD index: Right pulmonary artery distensibility index
RPAD: Right pulmonary artery in diastole
RPAS: Right pulmonary artery in systolic
EDV: End-diastolic volume
EDVI: End-diastolic volume index
ESV: End-systolic volume
ESVI: End-systolic volume index
RWT: Relative wall thickness
LVM: Left ventricular mass
LVMI: Left ventricular mass index
SV: Stroke volume
EF: Ejection fraction
MR: Mitral regurgitation
TR: Tricuspid valve regurgitation
CW: Continuous waves
E dec time: E wave deceleration time
TDI: Tissue doppler images
